# Pulmonary invasive mucinous adenocarcinoma mimicking pulmonary actinomycosis

**DOI:** 10.1186/s12890-022-01971-7

**Published:** 2022-05-06

**Authors:** Dongyi Zhu, Qian Zhang, Zhuanghua Rui, Shengbao Xu

**Affiliations:** Department of Respiratory Medicine, Shanghai East Hospital, Tongji University School of Medicine, Shanghai, 200123 China

**Keywords:** Pulmonary mucinous adenocarcinoma, Actinomycosis, CT-guided biopsy, Case report

## Abstract

**Background:**

Primary pulmonary invasive mucinous adenocarcinoma is a rare and distinct subtype of lung adenocarcinoma.

**Case presentation:**

A 72-year-old woman presented with productive cough for two months and fever for six days. Chest computed tomography (CT) showed a mass in the left lower lobe. Sputum culture tested negative for bacteria, but the sequence of *Actinomyces meyeri* was detected by metagenomic next generation sequencing from the bronchoalveolar lavage fluid. It was considered a pathogenic bacterium as the normalized number of DNA sequencing reads was 10 times higher than the normal level. The patient’s symptoms alleviated quickly, and the chest CT lesion shrank to a third of the original size following treatment with penicillin for two months. However, a repeat chest CT performed after four months of treatment revealed that the lesion had expanded. Positron emission tomography/CT revealed that fluorodeoxyglucose metabolism was increased in the mass with surrounding ground glass density of the left lower lobe. Furthermore, CT-guided percutaneous lung biopsy was performed, and hematoxylin–eosin staining showed columnar tumor cells with abundant mucin in the cytoplasm with a basal nucleus. Finally, the patient was diagnosed with pulmonary invasive mucinous adenocarcinoma and agreed to undergo a thoracoscopic surgery.

**Conclusions:**

Pulmonary invasive mucinous adenocarcinoma is a subset of lung adenocarcinoma with low incidence rate. The clinical features and CT findings are non-specific. A histopathological diagnosis is of fundamental importance in preventing misdiagnosis.

## Background

Primary pulmonary mucinous adenocarcinoma is a subtype of lung adenocarcinoma with low associated morbidity. It is classified into four types: mucinous adenocarcinoma in situ, minimally invasive mucinous adenocarcinoma, invasive mucinous adenocarcinoma (IMA), and colloid adenocarcinoma [1]. Altogether, IMA accounts for approximately 2–10% of lung adenocarcinomas; therefore, it is relatively uncommon [2]. It is often misdiagnosed as pneumonia due to a nonspecific computed tomography (CT) presentation, including consolidation, ground-glass opacities, and nodules. Herein, we report a case of a 72-year-old patient with cough for two months and fever for six days. Following penicillin treatment for presumed pulmonary actinomycosis, the lesion was initially reduced, but at four months it was increased again. Eventually, the patient was diagnosed with pulmonary IMA following CT-guided percutaneous lung biopsy. We present the following article in accordance with CAse REport (CARE) guidelines.

## Case presentation

**Fig. 1:**
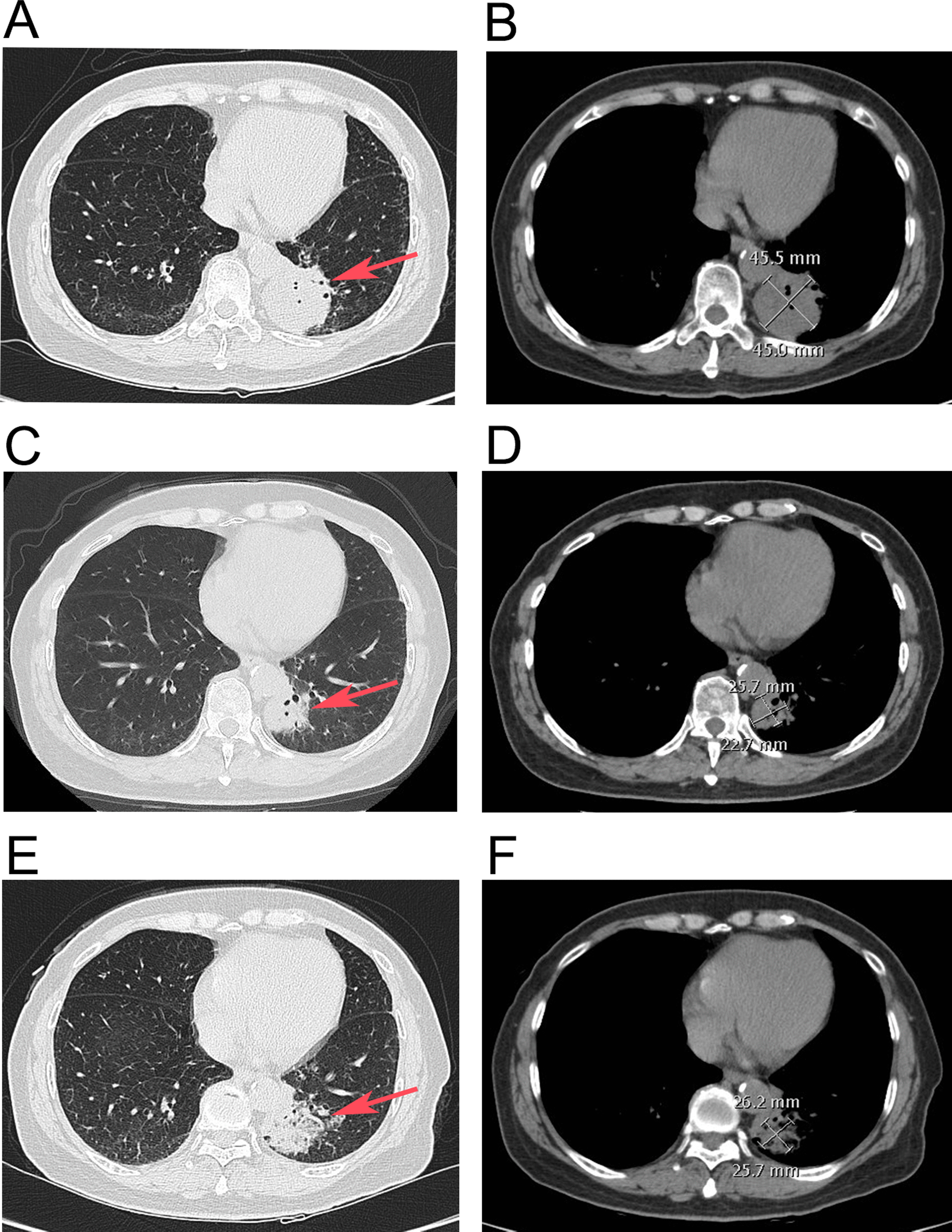
The change of chest computed tomography (CT) image. **a, b** The chest CT showing a mass (45.5 × 45.0 mm^2^) in the left lower lobe before treatment of penicillin. **c, d** The chest CT showing narrowed mass (25.7 × 22.7 mm^2^) in the left lower lobe after treatment of penicillin for 2 months. **e, f** The chest CT showing a mass (26.2 × 25.7 mm^2^) with expanded ground glass opacity in the left lower lobe after treatment of penicillin for 4 months. **a, c, e** pulmonary window, red arrow: mass; **b, d, f**: mediastinal window)

All procedures performed in studies involving human participants were in accordance with the ethical standards of the institutional and/or national research committee(s) and with the Helsinki Declaration (as revised in 2013). Written informed consent was obtained from the patient. The patient was a 72-year-old woman with productive cough for two months that she had ignored until she developed fever of 39 °C, particularly during the afternoon or night. The patient presented to a local hospital where laboratory test results showed elevated leukocytes (white blood cell count: 11 × 10^9^/L), neutrophils ratio (78%), and C-reactive protein level (30 mg/L). Chest CT showed a mass in the left lower lobe (Fig. 1a, b). The patient underwent bronchoscopy, and there were no abnormalities in any lumen. She was treated with cefoperazone/sulbactam and levofloxacin for four days with no effect. She had no additional symptoms, such as hemoptysis, night sweats, shortness of breath, or chest pain. She had no chronic diseases, such as hypertension, diabetes mellitus, heart disease, or family cancer history, while she was allergic to azithromycin.

As there was no response to treatment she was admitted to our hospital. Examination revealed moist rales on auscultation over the left lower lobe, while no lymph nodes were palpable. Routine blood test results were normal (white blood cell count, 5.77 × 10^9^/L; neutrophil ratio, 55.4%; hemoglobin concentration, 149 g/L; and platelet count, 205 × 10^9^/L) with a mildly elevated serum procalcitonin level (0.089 ng/mL). Liver and renal function tests were normal. Sputum for respiratory virus antigen, blood culture, serum 1–3-β-D-glucan and galactomannan were negative. The patient consented to a repeat bronchoscopy that showed congestion and edema of the bronchial mucosa in the left lower lobe. Bronchoalveolar lavage fluid (BALF) was collected for microbiological examination and metagenomic next generation sequencing (mNGS). A transbronchial lung biopsy (TBLB) was performed, and the tissue obtained underwent histopathological examination. The total cell count of the BALF was 115 × 10^6^/L, consisting of neutrophils (26%), macrophages (22%), lymphocytes (25%), and ciliated cells (26%). The mNGS showed that the normalized number (NN) of DNA sequencing reads for *Actinomyces meyeri* reached 115, which was more than 10 times the expected value. According to the Langelier’s study, the NN of DNA sequencing reads was used as the quantity for each microbe [3]. Species with NN less than three were removed, whereas species with NN greater than 10 were reported [3, 4]. Therefore, there was a high suspicion that this was a pathogenic bacterium. Meanwhile, the histopathological results of the TBLB reported normal tissue. Thus, the patient was diagnosed with pulmonary actinomycosis and received penicillin G 4 million units 8 hourly, intravenously for 14 days. Her body temperature returned gradually to normal, and her respiratory symptoms were relieved. The patient was discharged with amoxicillin 0.5 g orally three times daily, for six weeks. A repeat chest CT revealed that the lesion shrank to one-third of the original size (Fig. 1c, d). However, following a further two months of amoxicillin treatment, a third chest CT showed that the ground glass opacity appearance of the left-sided lesion had expanded compared with the previous CT (Fig. 1e, f). Pulmonary malignancy was suspected because of the lack of response to treatment.

**Fig. 2:**
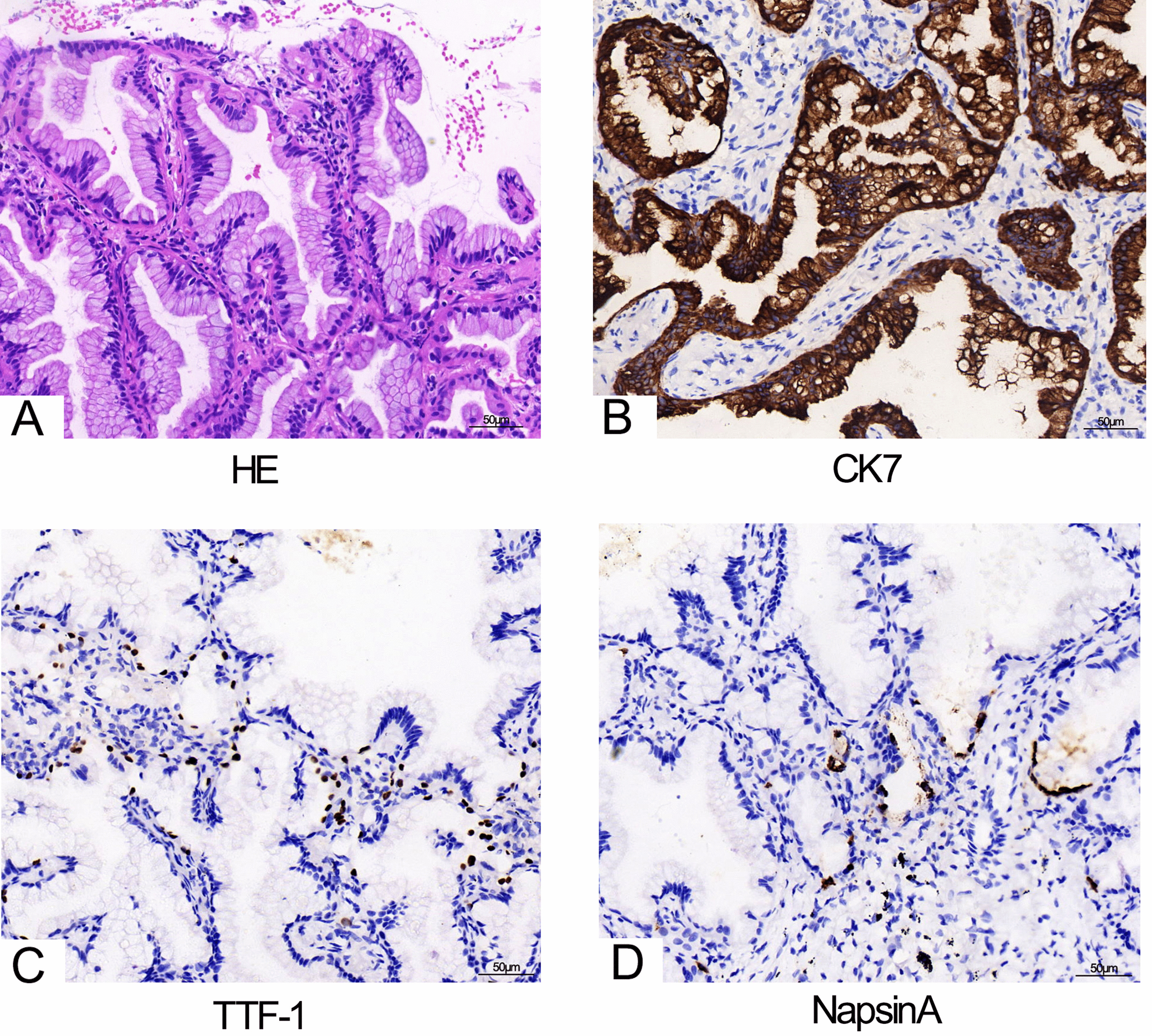
Pathological histology expression of lung. **a** A hematoxylin–eosin (HE) staining showing columnar tumor cells with abundant intracytoplasmic mucin and basally located nuclei (magnification × 200). **b–d** Immunohistochemistry staining showing the strong positive CK7 (**b**), partial positive Thyroid Transcription Factor-1 (TTF-1) (**c**) and the focal positive Napsin A (**d**) tumor cells (magnification × 200)

The patient was re-admitted to our hospital, and further examinations were performed. Positron emission tomography (PET)/CT revealed that fluorodeoxyglucose (FDG) metabolism was increased in the mass with surrounding ground glass opacities in the left lower lobe. The lesion area measured 65 × 63 mm^2^ with a standard uptake value (SUV) of 5.4. The patient consented to a CT-guided percutaneous transthoracic lung biopsy. Hematoxylin–eosin staining showed columnar tumor cells with abundant intracytoplasmic mucin and basally located nuclei (Fig. 2a). Immunohistochemical staining results showed CK7 (+), Thyroid Transcription Factor-1(TTF-1) (partial+), Napsin A (focal+), P40 (−), and CK20 (−), suggesting primary pulmonary IMA (Fig. 2b–d). Following these results, the patient was diagnosed with pulmonary IMA. Fortunately, the patient had no metastasis to lymph nodes or other organs. Eventually, the patient underwent thoracoscopic surgery, and the tumor was resected.

## Discussion and conclusions

Pulmonary IMA is an uncommon subtype of lung adenocarcinoma, accounting for 2.2–3.9% of resected adenocarcinomas [5]. The most frequent symptoms of IMA are sputum production, cough, shortness of breath, and fever, which are nonspecific. Typical chest CT findings usually include multicentric opacities or consolidation, ground-glass opacities, and nodules, commonly located in the lungs’ lower lobes [6, 7]. These clinical features mimic pneumonia. Morichika et al*.* reported a case of a 68-year-old man with a two-month history of coughing and sputum production. He was diagnosed with organizing pneumonia associated with *Mycobacterium fortuitum* infection by the BLAF culture. However, the surgical biopsy specimen was diagnostic for IMA, with no mycobacterial infection [8]. Beom et al. reported a case of a 64-year-old woman with a six-month history of cough, febrile sensation, and shortness of breath. She was diagnosed with chronic eosinophilic pneumonia by the elevated eosinophils. Despite administration of a systemic steroid, she did not rapidly respond. She was diagnosed as IMA through percutaneous needle biopsy [9]. In our case, the patient had high fever and cough with a mass in the left lower lobe on the chest CT. However, a bronchoscopy showed no atypical cells, though *A. meyeri* was detected in the BALF by mNGS.

Actinomyces are facultative anaerobic gram-positive bacteria. Their shape is filiform, and their growth slow [10]. *A. meyeri* is an uncommon cause of actinomycosis, and was first isolated from lung empyema in 1911 [11]. *A. meyeri* is likely to cause systemic dissemination, especially to the skin, liver, brain, long bones, and muscles. This unusual feature is mainly related to aspiration pneumonia [12]. Pulmonary actinomycosis is a great imitator and is often misdiagnosed as malignancy or tuberculosis. Thus, Kim et al*.* reported that 35.1% of such patients were initially diagnosed with lung cancer based on clinical and radiological findings [13]. The characteristic imaging on chest CT is consolidation, atelectasis, ground glass opacity, and pleural effusion. Gradually, the center of the lesion liquefies and becomes necrotic, leading to the formation of a cavity [14]. In this case, the left-sided lesion on the chest CT did not show liquefaction necrosis, which did not accord with the disease development. Furthermore, the ground-glass opacities around the mass expanded despite the four-month penicillin treatment.

The diagnosis of pulmonary actinomycosis depends on the visualization of actinomycete hyphae and sulfur granules on histopathological examination. In this case, although the mNGS of the BALF showed a high NN of DNA sequencing reads of *A. meyeri*, it was still difficult to be considered as a pathogen. It is difficult to detect anaerobic bacteria from sputum smears and cultures [10]. Furthermore, hematoxylin–eosin staining showed columnar tumor cells with abundant intracytoplasmic mucin but without actinomycete hyphae and sulfur granules, suggesting IMA was misdiagnosed as pulmonary actinomycosis and not combined with actinomycosis infection. The mass shrank observed by the administration of antibiotics might be due to the treatment for obstructive pneumonia caused by tumor compression. The obstructive pneumonia improved after antibiotic treatment.

Using PET/CT is of little value for the diagnosis of IMA. It does not really help in this differential diagnosis as pneumonia would be "hotter" than IMA. Erdoğan et al*.* revealed that the SUVmax on PET/CT may be high in benign conditions, such as organizing pneumonia (mean SUVmax, 6.5) [15]. Chang et al*.* revealed lower peak SUVs in IMA than in squamous cell carcinomas, non-mucinous adenocarcinomas, and other malignancies [16]. In this case, PET/CT showed an increased SUV (SUVmax 5.4) of the left mass. Uptake of FDG correlates directly with the number of cancer cells, but IMAs contain an abundant mucinous component with relatively low active cancer cells, which may account for this scant FDG uptake on PET/CT [17]. Lee et al*.* reported a higher FDG uptake (SUVmax, mean and standard deviation, 4.6 ± 3.9) in a consolidative pattern than in a nodular pattern (SUVmax, mean and standard deviation, 2.8 ± 2.8), and revealed that SUVmax was one of the independent predictors for poor disease-free survival in patients with IMA [18].

To obtain lung tissues for diagnosis, invasive operations need to be performed, including thoracoscopic or open lung biopsy, CT-guided percutaneous lung biopsy, and TBLB. Liu et al*.* reported that surgical lung biopsy and CT-guided percutaneous lung biopsy had a diagnostic yield of 100%, whereas TBLB combined with bronchoalveolar lavage had a diagnostic yield of 80.9% [19]. Thus, CT-guided percutaneous lung biopsy appears a more effective way to obtain diagnostic tissue, compared with TBLB; however, biopsied specimens could be composed of acellular mucin pools only because the alveolar spaces at the tumor periphery are often filled with mucin [2]. In this case, the histopathological result of the TBLB was negative for tumor cells. The BALF was collected for mNGS, but not for exfoliated cells, which may be one of the causes of misdiagnosis. Eventually, the patient was diagnosed with IMA using CT-guided percutaneous lung biopsy. As previous studies have revealed the limitations of CT-guided percutaneous lung biopsy, PET/CT should be performed beforehand for the puncture site to be guided by the SUVmax value.

In conclusion, pulmonary IMA has a low associated morbidity, with non-specific CT findings, often mimicking pneumonia. In contrast, pulmonary actinomycosis is often misdiagnosed as lung cancer. Therefore, a histopathological diagnosis is of great importance to prevent misdiagnosis.

## Data Availability

The datasets used and analysed during the current study are available from the corresponding author on reasonable request.

## Abbreviations

IMA: Invasive mucinous adenocarcinoma
CT: Computed tomography
BALF: Bronchoalveolar lavage fluid
mNGS: Metagenomic next generation sequencing
TBLB: Transbronchial lung biopsy
NN: Normalized number
PET: Positron emission tomography
FDG: Fluorodeoxyglucose
SUV: Standard uptake value
